# 血液肿瘤免疫及靶向药物治疗相关性感染预防及诊治中国专家共识（2021年版）

**DOI:** 10.3760/cma.j.issn.0253-2727.2021.09.002

**Published:** 2021-09

**Authors:** 中华医学会血液学分会感染学组 中华医学会血液学分会淋巴细胞疾病学组 中国临床肿瘤学会（CSCO）抗淋巴瘤联盟

血液系统恶性肿瘤如白血病和淋巴瘤等患者接受联合化疗后往往会出现骨髓抑制、中性粒细胞减少，合并感染是临床医师面临的难题。近年来，各种新疗法包括免疫治疗和分子靶向药物等在血液肿瘤中的应用越来越普遍，这些新疗法的作用机制与传统化疗不同，虽然骨髓抑制毒性较轻，但对机体免疫细胞和免疫功能具有独特的、不同程度的影响。免疫治疗和分子靶向药物导致的感染日益受到重视。美国国家综合癌症网络（NCCN）和欧洲白血病感染协作组（ECIL）针对当前应用的免疫治疗和分子靶向药物导致的相关感染并发症制定了诊断与鉴别诊断、预防与治疗管理专家共识[1]–[2]。中华医学会血液学分会与中国临床肿瘤学会（CSCO）抗淋巴瘤联盟基于国际共识、结合相关研究进展和国内情况制定了本共识，以规范和指导免疫及靶向药物治疗相关性感染的处理。

一、免疫治疗药物相关的感染

免疫治疗药物包括抗CD20单抗、抗CD38单抗、免疫检查点抑制剂、抗CD30单抗、双特异性抗体（BiTE）、抗CD79b单抗等已经上市和即将在国内上市的抗体药物（表1）。

**表1:** 抗体药物相关感染

类别	免疫系统影响	代表药物	感染事件
抗CD20单抗	①B细胞减少相关低丙种球蛋白血症；②中性粒细胞减少	利妥昔单抗	肺炎（4％）、结核、PcP、HBV再激活、CMV、VZV、JC病毒相关PML
抗CD38单抗	①B细胞减少相关低丙种球蛋白血症；②中性粒细胞减少	达雷妥尤单抗	上呼吸道感染（2％～26％）、肺炎（8％～15％）、HBV再激活、VZV
PD-1单抗	药物没有增加感染的风险，但irAE需要合并使用类固醇类药物，导致潜伏性感染再激活	纳武利尤单抗、帕博丽珠单抗、特瑞普利单抗、信迪利单抗、替雷利珠单抗	合并使用免疫抑制药物致机会性感染（7.3％）、结核、组织胞浆菌病、李斯特菌病感染
抗CD30单抗	①影响T细胞亚群比例平衡；②短暂的剂量依赖性中性粒细胞减少	维布妥昔单抗	肺炎（10％）、PcP、HBV再激活、CMV、VZV、HSV（1％～10％）、JC病毒相关PML
双特异性T细胞结合抗体	①B细胞减少相关低丙种球蛋白血症；②中性粒细胞减少	博纳吐单抗	治疗相关感染（45％，其中≥3级27％）、真菌感染罕见
抗CD79b单抗	中性粒细胞减少	Pola单抗	肺炎（4.4％）、PcP、CMV、JC病毒相关PML

注：irAE：免疫治疗相关不良反应；PcP：肺孢子菌肺炎；HBV：乙型肝炎病毒；CMV：巨细胞病毒；VZV：水痘-带状疱疹病毒；PML：多病灶脑白质病；HSV：单纯疱疹病毒；Pola单抗：Polatuzumab vedotin

（一）抗体药物对免疫系统影响

抗体药物的免疫抑制主要作用机制包括：①抑制B细胞免疫：外周血B细胞（包括CD19阴性浆细胞和CD19阳性浆母细胞）的快速及明显减少，可导致低丙种球蛋白血症，如BiTE、抗CD20单抗及抗CD38单抗[3]–[4]。②抑制T细胞免疫：如抗CD30单抗，通过杀死CD30阳性细胞，诱导免疫失调；通过细胞旁作用，杀伤体内T淋巴细胞（尤其是CD8^+^细胞），严重影响T细胞亚群比例平衡，导致感染发生。③治疗相关中性粒细胞减少：免疫治疗中普遍存在。④合并免疫抑制剂增加感染风险：如PD-1单抗常可引起免疫治疗相关不良反应（irAE），后者通常需要使用免疫抑制剂，这将进一步增加患者对致病做生物的易感性[5]。

（二）治疗相关感染的流行病学

1. 抗CD20单抗：利妥昔单抗（Rituximab）单药治疗时，≥3级中性粒细胞减少症发生率为4.2％～10.0％，感染以轻度和中度为主，常见的严重感染是肺炎（4％）。肺孢子菌肺炎（PcP）、巨细胞病毒（CMV）、水痘-带状疱疹病毒（VZV）、结核、严重西尼罗脑炎和巴贝斯虫病亦有报道。也可能引发合并JC病毒感染导致的多病灶脑白质病（PML）和乙型肝炎病毒（HBV）激活[4],[6]。

2. 抗CD38单抗：达雷妥尤单抗（Daratumumab）单药治疗主要引起<3级的上呼吸道感染（21％）[7]；与化疗药和（或）靶向药联合应用增加感染风险[8]–[10]，≥ 3级中性粒细胞减少症发生率为12％～75％，≥3级感染发生率为23％～28％，上呼吸道感染发生率为2％～26％，肺炎发生率为8％～15％，复发难治患者较初治患者感染风险高。另外，治疗期间易出现VZV感染和HBV再激活[11]。

3. 免疫检查点抑制剂：主要包括PD-1/PD-L1抗体和CTLA-4抗体等。PD-1单抗引起的irAE常需要使用免疫抑制剂，这将导致机会性感染，发生率可达7.3％[5]。结核、组织胞浆菌病和李斯特菌病也有报道[12]。

4. 抗CD30单抗：维布妥昔单抗（Brentuximab vedotin，BV）单药治疗时，≥3级中性粒细胞减少发生率为29％[13]。联合AVD方案时，其发生率增加到58％，且在第1个疗程时中性粒细胞减少性发热的发生率可达9％[14]。常见感染为肺炎（10％）[15]。VZV和单纯疱疹病毒（HSV）感染发生率为1％～10％，PcP发生率为0.1％～1％[16]，易出现CMV[17]和HBV[18]再激活。JC病毒感染所致的PML需高度警惕，可诱发死亡。

5. BiTE：博纳吐单抗（Blinatumomab）单药治疗时，≥3级中性粒细胞减少发生率为18％～32％，治疗相关感染发生率为45％，其中≥3级感染发生率为27％[19]。真菌感染较为罕见。长期连续输注期间（2～4周）需关注静脉导管相关感染。

6. 抗CD79b单抗：Polatuzumab vedotin（简称Pola单抗）单药治疗时，≥3级中性粒细胞减少症发生率为40％，肺炎发生率为4.4％；联合苯达莫司汀加利妥昔单抗治疗时，≥3级中性粒细胞减少发生率达46.2％，中性粒细胞减少性发热发生率为43.6％，≥ 3级感染发生率为23.1％，严重致命性感染发生率为10.2％[20]–[21]。严重感染包括败血症、肺炎（包括PcP和其他真菌性肺炎）、疱疹病毒和CMV感染。

三、分子靶向药物相关性感染

血液肿瘤常用靶向药包括酪氨酸激酶抑制剂（TKI）、蛋白酶体抑制剂（PI）、布鲁顿酪氨酸激酶（BTK）抑制剂、组蛋白去乙酰化酶（HDAC）抑制剂、JAK激酶抑制剂、BCL-2抑制剂、FLT3抑制剂和磷脂酰肌醇-3激酶（PI3K）抑制剂等（表2）。

**表2:** 分子靶向药物相关性感染

类别	免疫系统影响	代表药物	感染事件
TKI	①抑制非靶向激酶，造成CD4^+^和CD8^+^细胞增殖受抑制；②抑制B细胞功能；③中性粒细胞减少	伊马替尼、达沙替尼、尼洛替尼	肺炎；PcP；结核；HBV再激活；CMV；VZV；EBV
蛋白酶体抑制剂	①选择性耗竭T细胞；②中性粒细胞减少	硼替佐米、伊沙佐米	肺炎（5.0％～8.8％）；VZV感染（7.1％～22.3％）。流感住院率66.7％，重症监护病房住院率为41.6％
BTK抑制剂	①抑制B细胞发育，低丙种球蛋白血症；②抑制Toll样受体介导的感染	伊布替尼、泽布替尼、奥布替尼	最常见为上呼吸道感染，严重感染为肺炎；中枢真菌感染；HBV再激活；PcP；VZV；EBV。感染风险可随靶点特异性增加而下降
HDAC抑制剂	抑制Toll样受体介导的树状突细胞和巨噬细胞功能（传感、吞噬、细胞因子产生、黏附）	西达本胺	均见于联合用药，感染轻中度并可控
JAK激酶抑制剂	①抑制树突状细胞及CD4^+^ T细胞功能，降低Treg数量；②抑制NK细胞	芦可替尼	细菌感染（78％）；病毒感染（11％）；真菌感染（2％）；HBV再激活；PcP；VZV；CMV。（感染发生率随治疗时机延迟增加）
BCL-2抑制剂	中性粒细胞减少	维奈克拉	≥3级感染（淋巴瘤治疗：17.7％～19.0％；髓系肿瘤治疗：72％～74％）；IFD（19％，≥3级发生率为8％）
FLT3抑制剂	中性粒细胞减少	吉瑞替尼	≥3级中性粒细胞减少性发热（40.7％～66.3％）；肺炎（1.2％～13.9％）
PI3K抑制剂	①作用于T细胞和NK细胞，易引起免疫抑制；②中性粒细胞减少	Copanlisib	肺炎（常见，严重肺炎发生率为13.9％）

注：TKI：酪氨酸激酶抑制剂；BTK：布鲁顿酪氨酸激酶；HDAC：组蛋白去乙酰化酶；PI3K：磷脂酰肌醇-3激酶；Treg：调节性T细胞；PcP：肺孢子菌肺炎；HBV：乙型肝炎病毒；CMV：巨细胞病毒；VZV：水痘-带状疱疹病毒；EBV：EB病毒；IFD：侵袭性真菌病

（一）分子靶向药物对免疫系统影响

分子靶向药物所导致的感染风险增加是由于其对免疫系统功能的影响，根据不同作用位点，分别有以下几种机制：

1. 抑制B细胞：①BTK能使BCR通路异常激活，从而影响B淋巴细胞发育、分化和信号传导[22]；BTK基因突变可引起成熟B细胞发育缺陷及低丙种球蛋白血症[23]，从而造成过继免疫功能异常。②TKI也会抑制B细胞功能。

2. 抑制T细胞和NK细胞：①选择性PI3K抑制剂作用于T细胞和NK细胞，抑制免疫。②JAK激酶抑制剂抑制CD4^+^ T细胞激活和分化，减少Th1、Th17和调节性T细胞（Treg）数量[24]；抑制NK细胞活化和成熟[25]，从而增加感染风险。③PI可降低T细胞计数，下调Th1/Th2比例，抑制细胞毒性T细胞反应，导致T细胞亚群失衡，病毒再激活的风险增加，尤其是VZV[26]。④TKI以剂量依赖的方式抑制非靶点激酶活性，从而抑制CD4^+^和CD8^+^ T细胞增殖。通过抑制SRC激酶家族成员LCK，使T细胞受体上基于酪氨酸的免疫激活基序磷酸化，干扰T细胞活化并破坏CMV-/EB病毒（EBV）-特异性CD8^+^T细胞反应[27]。

3. 抑制先天性免疫：BTK及HDAC抑制剂对先天性免疫，特别是Toll样受体（TLR）介导的树突状细胞（DC）和巨噬细胞功能具有抑制作用[28]–[29]，以削弱炎症反应，并可能增加患者感染的风险。

4. 治疗相关中性粒细胞减少：靶向治疗中普遍存在。

（二）治疗相关感染的流行病学

1. TKI：伊马替尼（Imatinib）导致病毒及细菌感染的总发生率为14％，其中肺炎发生率为2％～4％，VZV感染或再激活率为2.0％～7.0％，感染主要发生在中性粒细胞减少期，特别是慢性髓性白血病（CML）慢性期晚期[30]–[31]。尼洛替尼（Nilotinib）相关感染的数据很少，其感染发生率为7.9％。达沙替尼（Dasatinib）治疗相关感染风险最高，总发生率为51％，其中肺炎和软组织感染最常见，治疗3个周期后风险逐渐上升，急性淋巴细胞白血病（ALL）患者或接受大剂量皮质类固醇治疗时感染风险更大[32]。TKI治疗相关机会性感染，包括EBV、结核、诺卡菌病、PcP、微小病毒B19感染和CMV再激活均有报道[33]–[34]，HBV再激活也多有报道[35]–[36]。

2. PI：PI治疗相关中粒细胞减少发生率为18.0％～21.4％，≥3级发生率为7.1％～17.0％[37]。最常见感染为肺炎、VZV和流感。肺炎发生率为5.0％～8.8％[37]–[38]，VZV感染发生率高达7.1％～22.3％[37],[39]。流感住院率高达66.7％，重症监护病房住院率为41.6％、死亡率达33.3％[40]。另外，机会性感染如诺卡菌病或者原藻病、PcP感染也有报道[41]。

3. BTK抑制剂：伊布替尼（Ibrutinib）一线治疗时，≥3级感染发生率为13％～36％，复发/难治患者为24％～51％。最常见为上呼吸道感染、泌尿道感染和鼻窦炎，严重感染以肺炎最常见（25％）[42]–[43]。此外，侵袭性真菌病（IFD）、隐球菌感染、VZV病毒再激活、PcP和EBV驱动的噬血细胞综合征均有报道[44]–[46]。6个月内感染发生率最高，随后明显降低，这可能与治疗后体液免疫和正常B细胞群的重建及稳定有关[42],[47]。新型BTK抑制剂优化了药物分子结构，提高了靶点特异性，降低了脱靶效应，感染发生率有下降趋势。泽布替尼（Zanubrutinib）治疗≥3级感染发生率为21.3％，奥布替尼（Orelabrutinib）治疗≥3级感染发生率仅为15.4％[48]。BTK抑制剂治疗后均可造成HBV再激活，发生率为1.0％～8.1％[49]。

4. HDAC抑制剂：西达本胺（Chidamide）是我国自主开发的HDAC抑制剂，其治疗相关不良事件大多为轻中度并可控，中性粒细胞减少发生率为22％，≥3级不良事件发生率为10％，均见于联合用药[50]–[51]。

5. JAK激酶抑制剂：芦可替尼（Ruxolitinib）治疗时，感染发生率为22％，其中≥3级感染占45％；细菌感染占78％，病毒感染占11％，真菌感染占2％。研究者发现，脾肿大伴国际预后评分系统（IPSS评分）中危-2及以上的患者发生的感染似乎更严重[52]。最常见尿路感染（9％）和VZV感染（1.9％）。5年随访数据显示，VZV感染发生率可随治疗时间延长而增加达11.5％，尿路感染可达24.6％，肺炎13％。败血症发生率为7.9％。重症感染少见，≥3级的VZV感染发生率为4.3％，≥3级尿路感染发生率为1.0％[53]–[54]。

6. BCL-2抑制剂：维奈克拉（Venetoclax）治疗难治复发淋巴瘤时，≥3级中性粒细胞减少发生率为57.7％，≥3级感染发生率为17.7％～19.0％[55]–[56]。治疗难治复发髓系肿瘤时，中性粒细胞减少症发生率为98％～100％，≥3级感染发生率为72％～74％，IFD发生率为19％，其中≥3级发生率为8％[57]–[58]。

7. FLT3抑制剂：吉瑞替尼（Gilteritinib）是我国首个获批用以治疗难治复发急性髓系白血病的FLT3抑制剂。单药治疗引起≥3级中性粒细胞减少性发热发生率为40.7％，严重肺炎发生率为1.2％[59]；与化疗药物和（或）靶向药物（维奈克拉）联合增加感染风险[60]–[61]，≥3级中性粒细胞减少性发热发生率为48.7％～63.3％，严重肺炎发生率为13.9％。另外，治疗期间IFD发生率为4％～25％[62]–[63]。

8. PI3K抑制剂：PI3K抑制剂单药引起≥3级中性粒细胞减少症发生率为23％～25％，最常见感染的是肺炎、败血症和中性粒细胞减少性发热，其中致命和（或）严重感染发生率为17.2％～21.0％[64]–[65]。

四、感染筛查与诊断

（一）治疗前感染筛查

根据不同的靶向和免疫治疗进行治疗前感染筛查（筛查重点参考表1～2）：①HBV、丙型肝炎、梅毒、获得性免疫缺陷综合征；②EBV、CMV、VZV、多瘤病毒；③红细胞沉降率；④结核分枝杆菌（结核特异性细胞免疫三项、TB SPOT、PPD试验等）；⑤呼吸道相关病毒（有上呼吸道症状者）；⑥半乳甘露聚糖试验/1,3-β-D-葡聚糖试验（GM/G试验）。

（二）感染的诊断

建议参考《中国中性粒细胞缺乏伴发热患者抗菌药物临床应用指南（2020年版）》[66]。

1. 病史询问和体格检查：详细了解既往靶向和免疫治疗药物情况、抗生素使用和定植情况，发现感染的高危和隐匿部位。

2. 实验室检查：全血细胞计数、肝肾功能和电解质检查、免疫球蛋白；降钙素原、C反应蛋白、IL-6等感染相关指标的检查对诊断有提示意义。

3. 病原学检查：

（1）血培养［至少同时行两份血培养检查，如果存在中心静脉导管（CVC），一份血标本从CVC的管腔采集，另一份从外周静脉采集。无CVC者，应采集不同部位静脉的两份血标本进行培养，采血量为每瓶10 ml。如果经验性抗菌药物治疗后患者仍持续发热，可以每2～3 d进行1次重复培养］。

（2）痰培养、粪培养、尿培养、脑脊液培养（必要时）。

（3）HBV、EBV、CMV、VZV、多瘤病毒。

（4）呼吸道相关病毒（有上呼吸道症状者）。

（5）GM/G试验。

（6）结核分枝杆菌（结核特异性细胞免疫三项、TB SPOT、PPD试验等）。

（7）支气管镜检查，肺泡灌洗。

（8）聚合酶链反应（PCR）和宏基因组二代测序（mNGS）。

4. 影像学检查：

（1）胸部高分辨CT；

（2）头颅MRI；

（3）腹部B超或CT。

5. 免疫相关性肺炎与感染性肺炎鉴别：

（1）免疫治疗后出现肺部症状（包括新发咳嗽、胸闷气促或低氧血症等）的患者，应尽早完善胸部CT检查，若肺部影像学出现毛玻璃样、网格状影改变时，则高度怀疑为免疫相关性肺炎；根据《中国临床肿瘤学会（CSCO）免疫检查点抑制剂相关的毒性管理指南》[67]中的肺毒性分级管理，必要时停药，并开始激素治疗。

（2）二者临床表现有相似之处，但目前尚无特异性的标志物将二者明确区分开来；且irAE合并感染的情况时有发生，因此，对二者发生发展的预判及干预时机把握至关重要。

（3）若二者无法明确鉴别，建议糖皮质激素治疗同时联合抗菌药物。

五、预防与治疗：总体原则见图1。

**图1:**
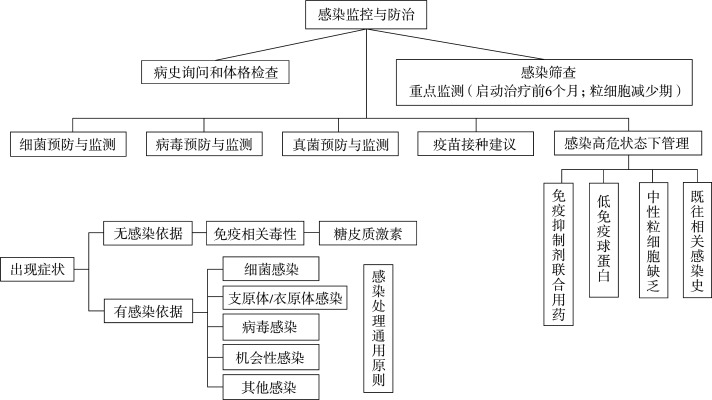
感染预防与监测总体原则

（一）感染预防与监测

1. 细菌预防与监测：目前尚无循证医学依据支持在靶向治疗同时予以常规细菌预防。启动靶向治疗后，根据临床情况，定期监测血常规、免疫球蛋白及降钙素原、C反应蛋白、IL-6等感染相关指标；部分药物感染事件主要发生于启动治疗后的前6个月，粒细胞减少期，需重点监测。

2. 病毒预防与监测：根据相关临床数据、国内外指南和药物说明，对不同类型免疫治疗和靶向药物治疗后HBV再激活[6]、VZV、CMV、EBV及JC病毒感染风险提出预防及监测建议，具体参见表3。

**表3:** 病毒预防与监测

类别	HBV	VZV	CMV	EBV	JC病毒
抗体药物相关病毒感染					
抗CD20单抗	推荐预防	推荐预防	推荐监测		警惕PML提示症状，如果确诊，则应永久终止治疗
抗CD38单抗	推荐预防	推荐预防			
抗CD30单抗	推荐预防	推荐预防			警惕PML提示症状，如果确诊，则应永久终止治疗
抗CD79b单抗		推荐预防	推荐监测		警惕PML提示症状，如果确诊，则应永久终止治疗
TKI	推荐预防	推荐预防	推荐监测	推荐监测	
蛋白酶体抑制剂	推荐预防	推荐预防			
BTK抑制剂	推荐预防	推荐预防	推荐监测	推荐监测	
HDAC抑制剂	推荐预防				警惕PML提示症状，如果确诊，则应永久终止治疗
JAK激酶抑制剂	推荐预防	推荐预防	推荐监测		
BCL-2抑制剂					警惕PML提示症状，如果确诊，则应永久终止治疗
PI3K抑制剂			推荐监测		

注：TKI：酪氨酸激酶抑制剂；BTK：布鲁顿酪氨酸激酶；HDAC：组蛋白去乙酰化酶；PI3K：磷脂酰肌醇-3激酶；HBV：乙型肝炎病毒；CMV：巨细胞病毒；VZV：水痘-带状疱疹病毒；CMV：巨细胞病毒；EBV：EB病毒；PML：多病灶脑白质病

3. 真菌预防与监测：根据相关临床数据、国内外指南和药物说明，对不同类型免疫治疗和靶向药物治疗相关的IFD及非典型真菌感染（如PcP）提出预防及监测建议，具体参照表4；部分药物相关感染事件主要发生于启动治疗后的前6个月，粒细胞减少期需重点监测。

**表4:** 真菌预防与监测

类别	IFD	PcP
抗体药物相关真菌感染		
抗CD20单抗	推荐监测	
抗CD38单抗	推荐监测	
PD-1单抗	若糖皮质激素（泼尼松≥20mg/d）应用≥6周，应考虑抗真菌预防	若合并糖皮质激素（泼尼松≥20mg/d）应用≥4周，应考虑预防PcP治疗
抗CD30单抗	推荐监测	推荐监测，但不常规预防
双特异性T细胞结合抗体	真菌感染罕见	
抗CD79b单抗	推荐监测	
分子靶向药物相关真菌感染		
TKI	真菌感染罕见	
蛋白酶体抑制剂	真菌感染罕见	
BTK抑制剂	推荐监测，特别是中枢性感染	推荐监测，但不常规预防
HDAC抑制剂	不良事件大多轻中度并可控	
JAK激酶抑制剂	推荐监测	推荐监测，但不常规预防
BCL-2抑制剂	推荐监测	
FLT3抑制剂	推荐监测	
PI3K抑制剂		治疗期间及停药后2~6个月常规预防[68]

注：IFD：侵袭性真菌病；PcP：肺孢子菌肺炎；TKI：酪氨酸激酶抑制剂；BTK：布鲁顿酪氨酸激酶；HDAC：组蛋白去乙酰化酶；PI3K：磷脂酰肌醇-3激酶；HBV：乙型肝炎病毒；CMV：巨细胞病毒；VZV：水痘带状疱疹病毒；CMV：巨细胞病毒；EBV：EB病毒。真菌预防：氟康唑400mg/d或泊沙康唑200 mg口服每日3次

4. 疫苗接种：国际文献建议应尽可能在开始抗肿瘤治疗之前给患者接种疫苗，包括水痘/带状疱疹、流感和肺炎疫苗等。应具体评估患者潜在的风险和收益做出选择，一般不建议接种减毒活疫苗，可以接种灭活疫苗（应答率可能下降）。肿瘤活动期通常不建议接种疫苗。特殊疫苗接种按国家相关规定。

5. 特殊感染监测：包括结核、组织胞浆菌病、李斯特菌病和诺卡菌病等；注意病史询问，特别是在常规抗感染药物治疗无效时，需考虑此类特殊病原菌感染可能。

6. 治疗期间特殊状态下感染管理建议：

（1）当ANC≤0.5×10^9^/L时：①可给予G-CSF 5～10 µg·kg^−1^·d^−1^；②高危中性粒细胞缺乏（粒缺）患者（如ANC≤0.1×10^9^/L或预计粒缺持续时间>7 d），推荐应用氟喹诺酮类药物抗细菌预防[66]；③推荐应用唑类药物初级抗真菌预防[69]（部分治疗药物与中强效CYP3A4抑制剂合并使用时需调整治疗剂量，详见表5；④必要时停用治疗药物，待ANC≥1.5×10^9^/L后，再次启动用药。

（2）对于IgG≤4 g/L（低丙种球蛋白血症）且合并严重或反复感染者，推荐静脉人免疫球蛋白（5 g×3 d）替代治疗。

（3）irAE发生后通常需要使用免疫抑制剂，若糖皮质激素（泼尼松≥20 mg/d）应用≥4周，应考虑PcP预防（复发磺胺甲恶唑800 mg每日2次，每周2次给药）；若糖皮质激素（泼尼松≥20 mg/d）应用≥6周，应考虑真菌预防（氟康唑400 mg/d或泊沙康唑200 mg每日3次）。具体参考《NCCN免疫治疗相关毒性的管理指南及CSCO免疫检查点抑制剂相关的毒性管理指南》[67],[71]。

（二）感染治疗原则（图2）

1. 感染类型：

（1）细菌感染：①一般细菌［革兰阳性菌（G^+^菌）、革兰阴性菌（G^−^菌）、厌氧菌］感染；②碳青霉烯类耐药非发酵菌感染。

（2）衣原体/支原体感染。

（3）病毒感染。

（4）机会性感染：①IFD；②分枝杆菌。

（5）其他：不明病原感染。

**图2:**
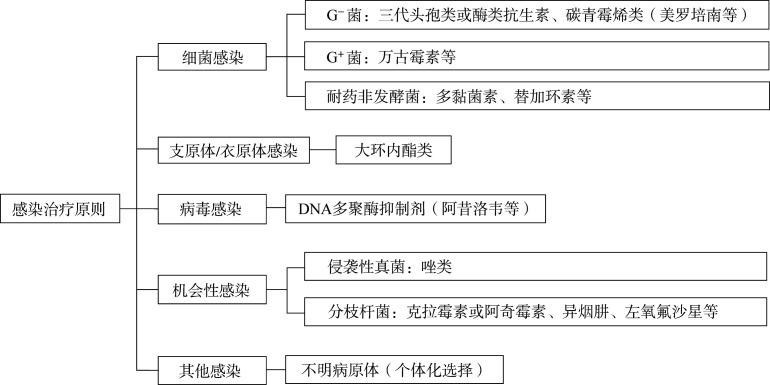
感染治疗原则（注意药物相互作用，特别是CYP3A4拮抗） G^−^菌：革兰阴性菌；G^+^菌：革兰阳性菌

2. 感染处理通用原则：考虑到免疫治疗及靶向药物治疗患者存在免疫功能抑制，故感染后应尽快启动抗菌药物初始经验治疗，而不必等微生物学的结果。抗感染治疗方案推荐如下［部分免疫治疗和靶向药与强效CYP3A4抑制剂有明显相互作用（表5），需暂时停药或者在密切监测下减量使用］：

（1）细菌感染：具体可参考《中国中性粒细胞缺乏伴发热患者抗菌药物临床应用指南（2020年版）》调整药物[66]：①G^−^菌：三代头孢类或酶类抗生素（如头孢曲松、头孢哌酮/舒巴坦、哌拉西林/他唑巴坦等），碳青霉烯类（如美罗培南、亚胺培南等）；②G^+^菌：万古霉素、替考拉宁、利奈唑胺等；③碳青霉烯类耐药非发酵菌：多黏菌素、舒巴坦及其复合制剂、替加环素。

（2）支原体/衣原体感染：大环内酯类，如阿奇霉素、红霉素等（大环内酯类抗生素为强效CYP3A4抑制剂，注意药物相互作用）。

（3）病毒感染：阿昔洛韦、伐昔洛韦、更昔洛韦、泛昔洛韦、膦甲酸钠。

（4）机会性感染：①真菌感染，具体可参考《血液病/恶性肿瘤患者侵袭性真菌病的诊断标准与治疗原则（第六次修订版）》[69]：伏立康唑、卡泊芬净、泊沙康唑和艾沙康唑。唑类抗真菌药物多为中强效CYP3A4抑制剂，治疗时需考虑到药物相互作用；艾沙康唑为CYP3A4弱抑制剂，药物相互作用最少，抗菌谱广；②分枝杆菌：克拉霉素或阿奇霉素、异烟肼、乙胺丁醇、利福平、左氧氟沙星、利福布汀等（注意CYP3A4拮抗）。

（5）严密监测患者感染的情况，并及时对药物剂量做出调整。

**表5:** 药物相互作用

类别	特殊药物合并使用时剂量调整建议^a^	
	强效CYP3A4抑制剂^b^	强效CYP3A4诱导剂
抗体药物		
抗CD20单抗	无	无
抗CD38单抗	无	无
PD-1单抗	无	无
双特异性T细胞结合抗体	增加未结合的MMAE AUC45%	降低未结合的MMAE AUC63%
抗CD79b单抗	无	无
分子靶向药物		
TKI	需减少给药剂量，监测用药反应	减低药物疗效，应避免同时使用
蛋白酶体抑制剂	无	无
BTK抑制剂	需减少给药剂量，监测用药反应	减低药物疗效，应避免同时使用
HDAC抑制剂	无	无
JAK激酶抑制剂	减少给药剂量约50%	无
BCL-2抑制剂	与泊沙康唑合用时，药物剂量需减少75%[70]（具体调整详见药物说明书）	减低药物疗效，应避免同时使用
FLT3抑制剂	需减少给药剂量，监测用药反应	减低药物疗效，应避免同时使用
PI3K抑制剂	减少Copanlisib剂量至45mg	减低药物疗效，应避免同时使用

注：TKI：酪氨酸激酶抑制剂；BTK：布鲁顿酪氨酸激酶；HDAC：组蛋白去乙酰化酶；PI3K：磷脂酰肌醇-3激酶；MMAE：一甲基澳瑞他汀E；AUC：曲线下面积。^a^部分免疫治疗和靶向药物与强效CYP3A4抑制剂合并使用时需调整剂量，如与泊沙康唑合用时，维奈克拉剂量至少应减少75％；使用强效CYP3A4抑制剂时，建议暂停使用伊布替尼或在密切监测下减量使用；与强效CYP3A4抑制剂同时用药时，芦可替尼每日总剂量应减少大约50％，并严密监视。^b^唑类抗真菌药物—强效CYP3A4抑制剂：酮康唑、伊曲康唑、泊沙康唑、伏立康唑；中效CYP3A4抑制剂：氟康唑；CYP3A4弱抑制剂：艾沙康唑。大环内酯类抗生素—强效CYP3A4抑制剂：克拉霉素、泰利霉素、氯霉素；中效CYP3A4抑制剂：红霉素。其他中效CYP3A4抑制剂：异烟肼

六、总结与展望

靶向治疗及免疫治疗开启了血液肿瘤治疗领域新格局，但其对于患者免疫系统的影响尚不十分清楚，特别是疾病进展或先前多次化疗的患者应用靶向及免疫治疗的潜在风险需要进一步评估。临床实践中应充分了解治疗相关感染的流行病学和发病机制，鉴别免疫相关的反应与感染，更好地处理免疫及靶向药治疗中相关性感染至关重要。本专家共识以期对临床实践有指导作用，并进一步修改完善。
